# Expanding metabolic pathway for de novo biosynthesis of the chiral pharmaceutical intermediate l-pipecolic acid in *Escherichia coli*

**DOI:** 10.1186/s12934-017-0666-0

**Published:** 2017-03-27

**Authors:** Hanxiao Ying, Sha Tao, Jing Wang, Weichao Ma, Kequan Chen, Xin Wang, Pingkai Ouyang

**Affiliations:** 1 State Key Laboratory of Materials Oriented Chemical Engineering, Nanjing, 211816 People’s Republic of China; 2 College of Biotechnology and Pharmaceutical Engineering, Nanjing Tech University, Nanjing, 211816 People’s Republic of China

**Keywords:** Chiral intermediate biosynthesis, Lysine cyclodeaminase, l-Pipecolic acid, Metabolic engineering, Cofactor engineering

## Abstract

**Background:**

The six-carbon circular non-proteinogenic compound l-pipecolic acid is an important chiral drug intermediate with many applications in the pharmaceutical industry. In the present study, we developed a metabolically engineered strain of *Escherichia coli* for the overproduction of l-pipecolic acid from glucose.

**Results:**

The metabolic pathway from l-lysine to l-pipecolic acid was constructed initially by introducing lysine cyclodeaminase (LCD). Next, l-lysine metabolic flux from glucose was amplified by the plasmid-based overexpression of *dapA, lysC,* and *lysA* under the control of the strong *trc* promoter to increase the biosynthetic pool of the precursor l-lysine. Additionally, since the catalytic efficiency of the key enzyme LCD is limited by the cofactor NAD^+^, the intracellular pyridine nucleotide concentration was rebalanced by expressing the *pntAB* gene encoding the transhydrogenase, which elevated the proportion of LCD with bound NAD^+^ and enhanced l-pipecolic acid production significantly. Further, optimization of Fe^2+^ and surfactant in the fermentation process resulted in 5.33 g/L l-pipecolic acid, with a yield of 0.13 g/g of glucose via fed-batch cultivation.

**Conclusions:**

We expanded the metabolic pathway for the synthesis of the chiral pharmaceutical intermediate l-pipecolic acid in *E. coli.* Using the engineered *E. coli,* a fast and efficient fermentative production of l-pipecolic acid was achieved. This strategy could be applied to the biosynthesis of other commercially and industrially important chiral compounds containing piperidine rings.

**Electronic supplementary material:**

The online version of this article (doi:10.1186/s12934-017-0666-0) contains supplementary material, which is available to authorized users.

## Background

**Fig. 1:**
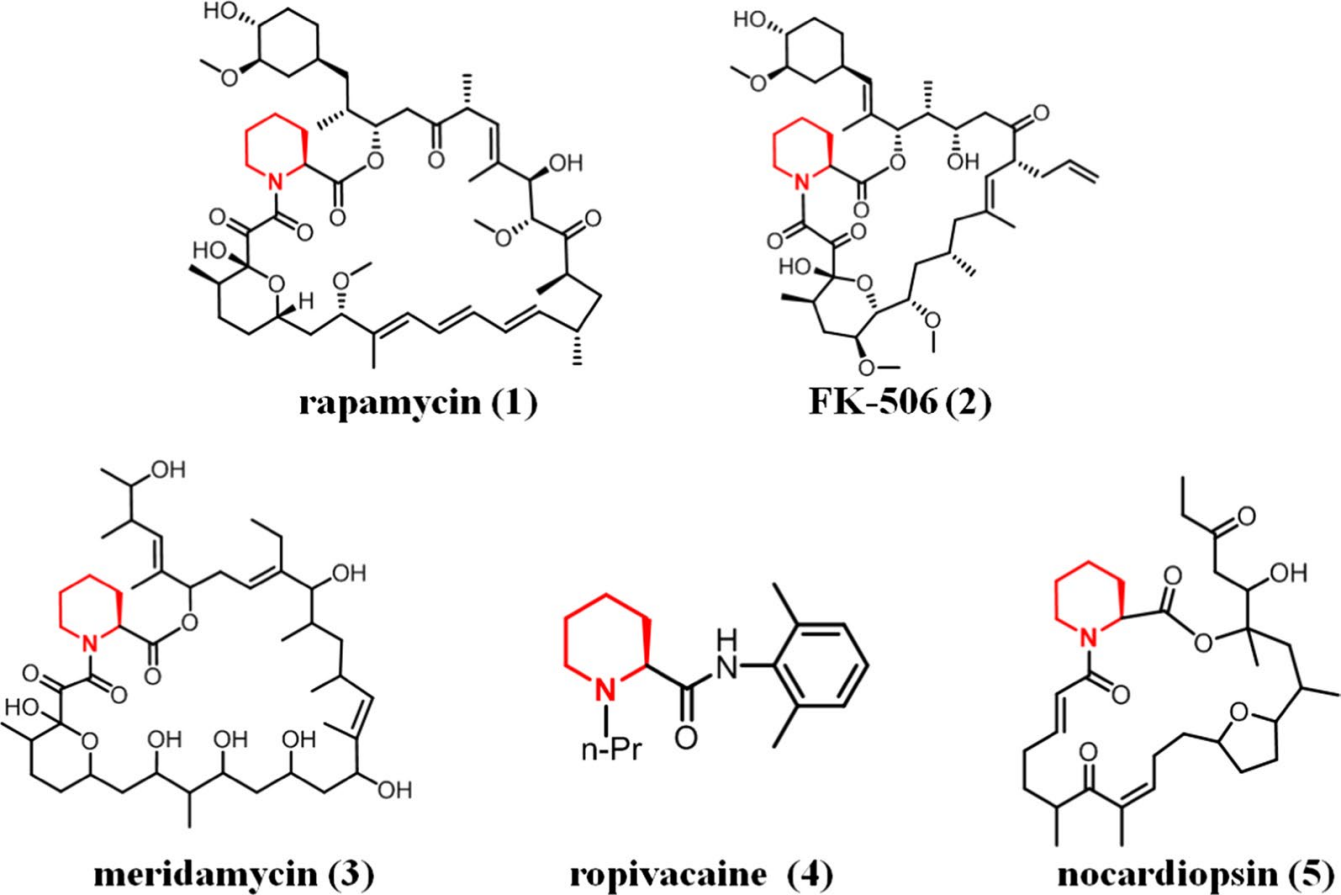
The structures of pipecolate derivatives. The chemical structures of pipecolate derivatives including (*1*) rapamycin, (*2*) FK-506, (*3*) meridamycin, (*4*) ropivacaine, (*5*) nocardiopsin. The pipecolate groups are represented in *red*

Chirality plays a key role not only in the life of plants and animals but also in the pharmaceutical, agricultural and chemical industries [1, 2]. Particularly, in the pharmaceutical industry, 56% of all drugs currently in use are chiral and screening and production of single enantiomers of chiral intermediates is increasingly important [3]. l-pipecolic acid (l-PA) is a non-proteinogenic compound and key chiral intermediate in the synthesis of many important groups of drugs [4] (Fig. 1). l-PA with a high optical purity is a precursor for the anaesthetics ropivacaine, bupivacaine and chloroprocaine which are widely used in local anaesthesia [5]; and it is also a precursor for the macrolide-pipecolate immunosuppressor rapamycin, FK-506 and FK-520 that are indispensable in the clinic [6]. Similar to the preparation of most of the other enantiomeric organic compounds, pure l-pipecolic acid enantiomers are mainly produced by chemical enantioselective synthesis [7] and stereoselective transformation [8]. However, the chemical methods for commercial-scale preparation of l-PA are complex and environmentally unfriendly [9].

Total biosynthesis of chiral drug intermediates is a more environmentally friendly approach for the preparation of affordable pharmaceuticals [10]. Bioproduction of l-PA has been extensively investigated, mainly due to its close relationship with l-lysine metabolism [11, 12]. Previous studies [11–13] have established two basic routes for converting l-lysine to l-PA. The first involves the loss of the α-amino group of l-lysine and incorporation of the ε-nitrogen into l-pipecolic acid (P2C pathway). The second involves the loss of the ε-nitrogen and incorporation of an α-nitrogen into l-PA (P6C pathway). Several studies on the enzymatic production of l-PA using enzymes from both pathways have been reported [14, 15]. For example, Neshich et al., obtained l-pipeclic acid on lysine containing medium (about 0.6 μg/mg protein) using recombinant *E. coli* cells over-expressing the l-lysine 6-dehydrogenase gene in P6C pathway [16]; Fujii et al. reported that about 3.9 g/L of optically pure l-PA was biosynthesised using the enzymes from the P2C pathway [17]. Recently, studies on the biosynthesis of rapamycin in *Streptomyces hygroscopicus* found that lysine cyclodeaminase (LCD) could directly catalyse the conversion of l-lysine to l-PA in one step [18]. In our previous studies, recombinant *E. coli* over-expressing LCD could produce l-PA from l-lysine with a yield of nearly 70% [19]. In comparison with the P2C and P6C pathways, the LCD pathway has many advantages, not least its simplicity, and this makes it more suitable for combining with complicated upstream metabolism processes.

The use of purified enzymes or cells as catalysts is often limited by high cost and recycling-associated problems [20]. Compared with traditional catalytic processes, fermentation can simplify the production procedures, potentially lowering cost and improving efficiency [21]. Very recently, Wendisch et al. described a fermentation method for the production of l-PA from glucose via the P6C pathway that include two enzymes and a spontaneous chemical reaction in *Corynebacterium glutamicum*, achieving a l-PA titer of 14 mM (1.73 g/L) [22]. Given the complexity if the l-PA biosynthesis pathway (P6C), it may be possible to improve this method considerably.

In the present study, we developed a new strategy for fermentative l-PA production from glucose. Recombinant *E. coli* expressing lysine cyclodeaminase was constructed followed by the increasing of the precursor l-lysine flux. In addition, the concentration of intracellular cofactors was rebalanced by overexpressing transhydrogenases, which improved the catalytic efficiency of the key lysine cyclodeaminase enzyme. Finally, optimization of the fermentation conditions resulted in the highest fermentative l-PA concentration yet reported using a fed-batch fermentation approach.

## Methods

### Strains and plasmids

Strains and plasmids are summarized in Additional file 1: Table S1. All engineered constructs were verified by colony PCR and Sanger sequencing. The detailed pathway construction procedure is described in the Additional file 1.

### Media and cultivation conditions

The *E. coli* seed cultures were grown in Luria–Bertani (LB) medium. The recombinant strains used to confirm l-lysine and l-pipecolic acid production ability were cultured in the minimal medium containing: 10 g/L glucose, 17.1 g/L Na_2_HPO_4_·12H_2_O, 0.5 g/L NaCl, 3.0 g/L K_2_HPO_4_, 10 g/L NH_4_Cl, 0.22 g/L CaCl_2_, 2.4 g/L MgSO_4_, and 0.01 g/L FeSO_4_. Recombinant strains used to investigate the effect of *pntAB* and fermentation supplements were cultured in the modified LB medium containing 5 g/L glucose, 5 g/L tryptone, 4 g/L yeast extract, and 10 g/L MOPS. Recombinant strains used for fed-batch fermentations were cultured in the medium containing 15 g/L glucose, 12 g/L tryptone, 8 g/L yeast extract, 2.1 g/L citric acid·H_2_O, 2.5 g/L (NH_4_)_2_SO_4_, 0.1 g/L FeCl_3_, 0.5 g/L MgSO_4_·7H_2_O, 0.5 g/L K_2_PO_4_·3H_2_O, 3 g/L KH_2_PO_4_, and 15.13 g/L Na_2_HPO_4_·12H_2_O. Appropriate antibiotics were added at the following concentrations: 50 μg/mL kanamycin, 34 μg/mL chloramphenicol and 40 μg/mL streptomycin. All media were heat-sterilized at 121 °C for 15 min.

A seed inoculum of 500 μL from an overnight 5 mL LB culture was added to a 500 mL flask containing 100 mL of corresponding medium for propagation at 37 °C with orbital shaking at 200 rpm. When the OD_600_ reached 0.6, IPTG (0.1 mM) was added to induce protein expression at 25 °C and growth continued for 48 h, after which the titer of l-lysine and l-PA was measured.

Fed-batch fermentation was carried out in a 7.5 L fermentor (BioFio 115, New Brunswick Scientific, Edison, NJ, USA) with an initial broth volume of 3 L. The culture pH was monitored by using a Mettler electrode and maintained at 7.0 with the addition of concentrated ammonium hydroxide (25% w/w NH_3_). The dissolved oxygen (DO) content was monitored using a Mettler oxygen electrode and was maintained at about ~15% by adjusting the agitation speed and aeration. Once the initial glucose was exhausted, glucose (700 g/L) and organic nitrogen sources (tryptone, 20 g/L, yeast extract, 15 g/L) feeding was initiated. The residual glucose concentration was monitored off-line by SBA-40C biosensor analyzer and maintained at 5 g/L by adjusting the pump feeding rate manually. IPTG (0.1 mM) was added when the OD_600_ reached 8–10, and culturing continued for 72 h. Samples were removed for measuring cell growth and the concentration of l-lysine and l-pipecolic acid every 2–12 h.

### Analytical methods

The DCW was computed from a curve of optical density measured at 600 nm (OD_600_) with respect to dry weight. An OD_600_ of 1.0 represented 45 mg dry weight per liter. The concentration of glucose and l-lysine was analyzed using a SBA-40C biosensor analyzer [23] (Shandong Province Academy of Sciences, Jinan, China) as previously described. The protein expression were examined by SDS-PAGE. Firstly, the cells were harvested and concentrated to OD_600_ = 10. After ultrasonic decomposition and centrifugation, the supernatant and the precipitation were mixed with protein loading buffer (Takara, Dalian, China), 99 °C denatured for 10 min for loading the gel.

The exact l-PA and l-lysine concentrations were determined using high-performance liquid chromatography after labelling with phenylisothiocyanate (PITC) [24], using a 1290 apparatus(Agilent Technologies, USA) equipped with a C18 column (5 μm, 250 mm × 4.6 mm, Grace, USA) at room temperature. Mobile phase A consisted of 7% (v/v) acetonitrile in 0.1 M sodium acetate aqueous solution, and mobile phase B consisted of 80% (v/v) acetonitrile in water. PITC derivatives were separated with a gradient of 97:3 to 30:70 (v/v) of A: B over 50 min and detected at an absorbance of 254 nm.

The intracellular NAD^+^, NADH, NADP^+^ and NADPH concentration was measured using dedicated quantification kits purchased from Sigma-Aldrich. The content of NAD^+^ and NADH bound to purified LCD was estimated using an HPLC method based on that described previously by Yuan and Borchardt [25]. A quantity of 200 µL of an 100 µM solution of purified LCD (20 nmol) was precipitated with 3 volumes of 95% ethanol. Supernatants were flash-frozen in liquid N_2_ and lyophilized for 12 h. The residue was dissolved in 100 µL of water and analysed by HPLC (Agilent 1290 Technologies, USA) using a C18 column (5 μm, 250 mm × 4.6 mm, Grace, USA) with monitoring at 260 and 340 nm. NAD^+^ and NADH were separated under isocratic conditions with 2.5% methanol in 0.1 M sodium phosphate buffer (pH 7.0) [18]. Standard curves were generated with authentic NAD^+^ and NADH to correlate peak area with the amount of cofactor.

## Results

### Construction of a metabolic pathway for conversion of l-lysine to l-PA

**Fig. 2:**
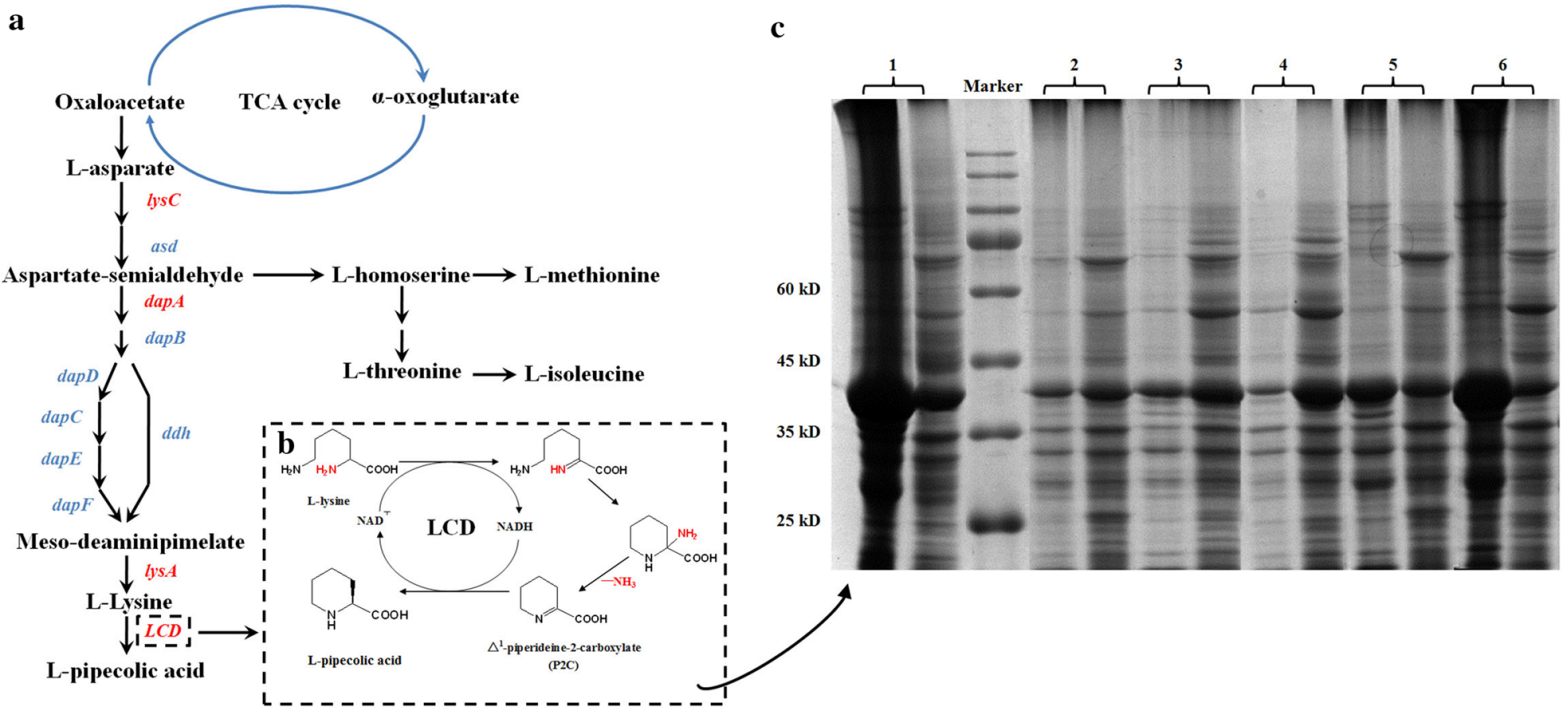
The metabolic pathway of l-pipecolic acid and the heterologous expression the key enzyme LCD. **a** The pathway and the strategies for metabolic engineering for l-pipecolic acid overproduction. The genes represented in* red* indicate increased flux by directly overexpression in the final recombinant strain *E. coli* BL21–*dapA*–*lysC*–*lysA*–*pntAB*–*pipA*. Enzymes encoded by the genes shown are: *asd*, aspartate semialdehyde dehydrogenase; *dapA*, dihydrodipicolinate synthase; *dapB*, dihydrodipicolinate reductase; *dapC*,* N*-succinyldiaminopimelate-aminotransferase; *dapD*, tetrahydrodipicolinate succinylase; *dapE*,* N*-succinyl-l-diaminopimelate desuccinylase; *dapF*, diaminopimelate epimerase; *ddh*, meso-diaminopimelate dehydrogenase from *C. glutamicum*; *lysA*, diaminopimelate decarboxylase; *lysC*, aspartate kinase III. **b** The detailed LCD catalytic process. The disassociated amine group are represented in red. **c** The heterologous expression of the key enzyme LCD. The over expressed LCD is about ~39 kD. *Lane group 1* 37 °C induced with 1 mM IPTG; *Lane group 2* 25 °C induced with 0.1 mM IPTG; *Lane group 3* 25 °C induced with 0.3 mM IPTG, *Lane group 4* 25 °C induced with 0.5 mM IPTG; *Lane group 5* 25 °C induced with 0.7 mM IPTG; *Lane group 6* 25 °C induced with 1 mM IPTG. The proteins in sediment is analyzed on the *left* while the protein in supernatant is analyzed on the *right* in every lane group

To expand the metabolic pathway and include l-pipecolic acid as a metabolite, we introduced a unique enzyme into the *E. coli* (Fig. 2a, b) [19]. The *pipA* gene from *Streptomyces pristinaespiralis* ATCC25486 encoding the lysine cyclodeaminase was amplified and cloned into the vector pET-28a to generate the recombinant plasmid pET-28a-*pipA* followed by the transformation to the *E. coli* BL21(DE3). Further, to investigate the catalytic activity of LCD, the cells were cultured, induced at 37 °C with 1 mM IPTG for 6 h and concentrated to 5 g_DCW_/L. The results showed that with an addition of 1 g/L l-lysine to the whole-cell *E. coli* biocatalyst and crude enzyme liquid extracted from same concentration of whole-cell biocatalyst, 0.24 and 0.15 g/L l-PA was obtained, respectively, which confirmed the ability of the engineered strain to convert l-lysine to l-PA.

However, this relatively low bioconversion rate indicated that the overexpression of LCD in *E. coli* was not optimal and SDS-PAGE analysis confirmed that a large fraction of the overexpressed enzyme was present in insoluble inclusion body form (Fig. 2c, Lane 1). To increase the soluble expression of LCD in *E. coli*, the induction temperature was lowered to 25 °C to avoid the rapid generation of the recombinant protein which might cause the insolubility. As shown in Fig. 2c Lane 6, slightly decrease in inclusion body was observed. Next, five different IPTG concentrations ranging from 0.1 to 1.0 mM were tested to induce the LCD expression (Fig. 2c Lanes 2–6), and 0.5 mM IPTG was optimal (~two to threefold higher than controls, Fig. 2c Lanes 1, 4). This change increased the bioconversion from l-lysine to l-PA to 45 and 41% using whole-cell and crude enzyme systems, respectively, which was 87.5 and 173% higher than before optimization. However, despite the successful overexpression and considerable enzymatic activity, l-PA was not detectable in shake flask fermentations using minimal media (Fig. 2c), possibly due to the absence of the direct precursor of LCD.

### Amplification of the l-lysine precursor metabolic flux

**Fig. 3:**
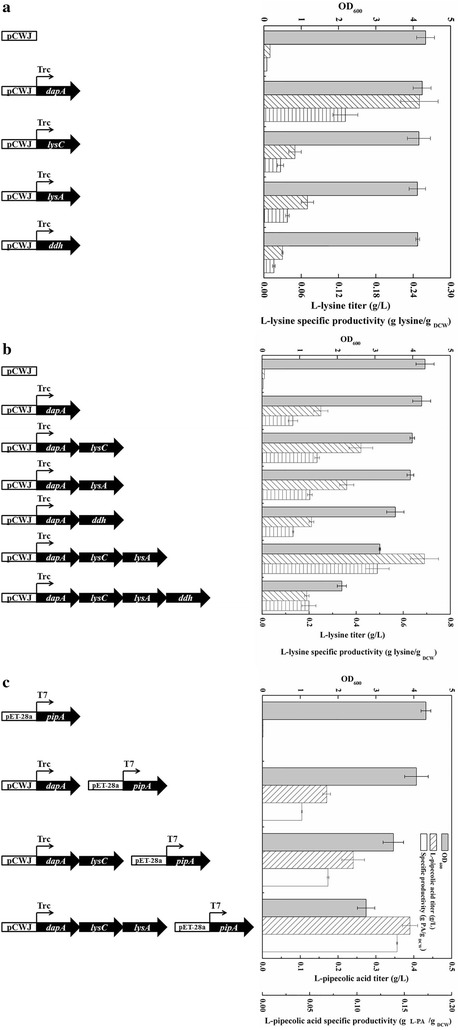
Gene screening for increasing the biosynthetic pool of precursor l-lysine. The concise descriptions of the genes and plasmid construction strategy are on the *left* with the corresponding results on the *right*. The *filled bar* represents OD_600_, the *bar with forward slash* represents l-lysine titer (g/L), the *bar with backslash* represents l-pipecolic acid titer (g/L), the *bar with horizontal lines* represents l-lysine specific productivity (glysine/g _DCW_), the *unfilled bar* represents l-pipecolic acid specific productivity (g l-PA/g _DCW_). **a** Screening of single genes for improving the l-lysine metabolic flux. **b** Screening of gene combinations for improving the l-lysine metabolic flux. **c** Screening of gene combinations for l-pipecolic acid overproduction

l-Lysine acts as the direct and the only precursor for l-PA, and therefore plays the key role in l-PA accumulation. To increase l-lysine production, four genes (*dapA*, *lysA*, *lysC*, and *ddh*) from l-lysine metabolic pathways in either *E. coli* or *C. glutamicum* were over-expressed in *E. coli* BL21(DE3) using pCWJ vectors under the control of the *trc* promoter. Strains harboring different plasmids were cultured in the shake flasks containing minimal media for 48 h. The results (Fig. 3a) showed that the growth of strains was not seriously affected by the over-expression of these four genes, since the OD_600_ value was decreased by less than 10%. Compared with the CK control strain, a dramatic increase in lysine titer was achieved upon over-expression of *dapA*, which reaches 0.24 g/L with a specific productivity (g lysine/g_DCW_) of 0.13. In addition, the *lysC* and *lysA* had a similar positive effect on the lysine accumulation, leading to the lysine titer of 0.05 and 0.07 g/L, respectively. However, the *ddh* gene from the *C. glutamicum* was less effective, with a lysine titer of only 0.03 g/L and a specific productivity of <0.02.

To further improve the production of the lysine, the combining over-expression of *dapA*, *lysC*, *lysA* and *ddh* were conducted. Based on the results of the single gene screening, *dapA* was considered the most essential gene for co-expression and five different combinations including *dapA*–*lysC*, *dapA*–*lysA*, *dapA*–*ddh*, *dapA*–*lysC*–*lysA* and *dapA*–*lysC*–*lysA*–*ddh* were over-expressed in BL21(DE3) using pCWJ vectors under the control of the *trc* promoter. Culturing of these strains in shake flasks with minimal media for 48 h resulted in a decrease in cell growth with an increasing number of genes over-expressed (Fig. 3b). The growth of recombinant strain BL21–*dapA*–*lysC*–*lysA*–*ddh* over-expressing all four genes was almost half that of CK. However, the growth of the other four strains remained over 70% that of CK, suggesting over-expressions of three or fewer genes had a slight negative effect on cell growth. Co-expression of *dapA*, *lysC* and *lysA* resulted in the highest lysine production, with a titer and specific productivity of 0.69 g/L and 0.49 (g/g_DCW_), respectively, 2.76-fold higher than the single over-expression strain BL21-*dapA*. In addition, lysine production by BL21–*dapA*–*lysC*–*lysA*–*ddh* was only 0.19 g/L, which was slightly lower than the BL21–*dapA*, suggesting the further over-expression of *ddh* without optimization of the whole biosynthesis pathway might lead to an unbearable burden for the host strain.

According to our previous studies, the LCD activity might be inhibited by excessive lysine [19], thus, the pET-28a–*pipA* was not only transformed into highest lysine production strain BL21–*dapA*–*lysC*–*lysA*, but also BL21–*dapA* and BL21–*dapA*–*lysC* that still produce a considerable amount of lysine. Recombinant strains were cultured in the shake flasks with minimal media for 48 h, and l-PA was biosynthesized in all strains provided the precursor lysine was produced (Fig. 3c), confirming the crucial role of the lysine pathway engineering in l-PA biosynthesis. In general, substrate inhibition did not occur at the l-lysine concentrations employed and the l-lysine was totally consumed in all strains. l-PA titer increased with the increasing lysine concentration up to 0.39 g/L, with a yield (relative to lysine) of 0.57 g/g in the strain BL21–*dapA*–*lysC*–*lysA*–*pipA*. Heterologous expression of LCD and improvement of the lysine flux were therefore successful for engineering *E. coli* to produce l-PA from glucose. BL21–*dapA*–*lysC*–*lysA*–*pipA* was the highest l-PA production strain, and was used in subsequent experiments.

### Rebalancing of the intracellular nicotinamide concentration by introducing PNTs

The metabolic pathway of l-PA production requires NAD^+^ as a cofactor for LCD. Although the NAD^+^ and NADH can be recycled in situ, the LCD can only be activated by the redox state of the nicotinamide (our unpublished work). In addition, According to Walsh et al., LCD was incompletely loaded with nicotinamide cofactors (NAD^+^ and NADH) by 50% when overproduced in *E. coli*, and only a third of molecules contained NAD^+^, suggesting LCD activity may be sensitive to the intracellular cofactor concentration [18].

**Fig. 4:**
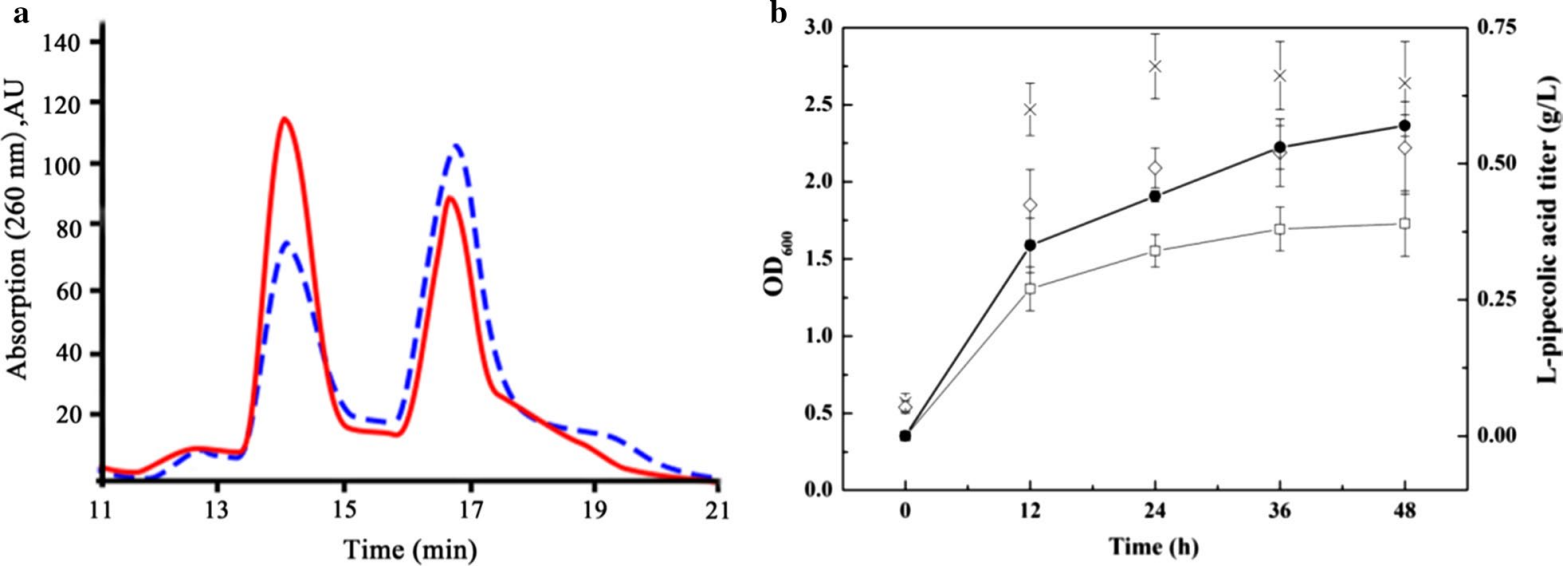
Effect of over-expressing PNTs on l-pipecolic acid production. **a** The HPLC analysis of the NAD^+^/NADH bound to LCD that over-expressed with PNTs or without PNTs. The *red line* represents the LCD that over-expressed with PNTs, the *blue dashed line* represents the LCD that over-expressed without PNTs. The peak at 14.2 min is NAD^+^ and the peak at 16.9 min is NADH. **b** The effect of over-expressing PNTs on l-pipecolic acid production. The *unfilled square* represents l-pipecolic acid titer of strain without over-expressing PNTs, the *filled circle* represents l-pipecolic acid titer of strain with over-expressing PNTs (g/L); the *cross* represents OD_600_ of stain without over-expressing PNTs, the unfilled diamond represents OD_600_ of stain with over expressing PNTs

A membrane-bound PNT transhydrogenase encoded by *pntAB* gene has been described previously and used for the regeneration of NAD^+^ and NADPH [26]. The PNT enzymes are composed of α and β subunits encoded by *pntA* and *pntB* genes that catalyze the reduction of NADP^+^ to NADPH via oxidation of NADH to NAD^+^: NADPH + NAD^+^⇌NADP^+^ + NADH [27]. Herein, PNTs were introduced into the BL21–*dapA*–*lysC*–*lysA*–*pipA* strain to rebalance the nicotinamides and enhance LCD activity. As summarized in Table 1, the intracellular concentration of NAD^+^, NADH, NADP^+^, NADPH and their ratios were measured in recombinant cells during the exponential growth phase. Although PNTs had tiny effect on the NADH/NAD^+^ ratio, it dramatically lowered the NADPH/NADP^+^ ratio by 37.8%, indicating a redistribution of the nicotinamide content in the engineered strains. Although no significant differences in total nicotinamides were observed between the two strains, the four nicotinamides were present at different concentrations. The presence of PNTs increased the NAD^+^ concentration remarkably by 22.7% and the NADH concentration increased slightly leading to a accumulation of NAD^+^/NADH pool. However, the NADPH concentration decreased slightly, while the NADP^+^ concentration remained at a relatively stable levels. An HPLC method used to separate NAD^+^ from NADH reported by Walsh et al. was applied to 20 nmol LCD purified from BL21–*dapA*–*lysC*–*lysA*–*pipA* and BL21–*dapA*–*lysC*–*lysA*–*pntAB*–*pipA*. As shown in Fig. 4a, an over-expression of PNTs significantly increased the concentration of NAD^+^ bound to LCD, and slightly decreased the NADH bound LCD concentration. To conclude, 0.23 mol NAD^+^/mol LCD and 0.29 mol NADH/mol LCD was obtained with the strains expressing PNTs, which is 24.3% higher and 8.4% lower than without expressing PNTs, respectively. Further, the recombinant strains were cultured in modified LB medium for 48 h after induction, and with the over-expression of PNTs, the l-PA titer was increased 35.9% compared with BL21–*dapA*–*lysC*–*lysA*–*pipA*, reached 0.53 g/L (Fig. 4b).

**Table 1:** Intracellular concentrations of NAD^+^, NADH, NADP^+^, and NADPH in recombinant cells during exponential growth

Strains	Intracellular concentrations (μmol/g_dcw_)					NADH/NAD^+^ ratio	NADPH/NADP^+^ ratio
	NAD^+^	NADP^+^	NADH	NADPH	Total		
PA	1.54 ± 0.04	0.14 ± 0.02	0.35 ± 0.02	0.73 ± 0.03	2.76 ± 0.05	0.22 ± 0.02	5.21 ± 0.05
PA-PNTs	1.89 ± 0.07	0.13 ± 0.01	0.39 ± 0.01	0.42 ± 0.03	2.83 ± 0.06	0.20 ± 0.01	3.23 ± 0.03

### Effect of Fe^2+^ and surfactant on l-PA fermentation

**Fig. 5:**
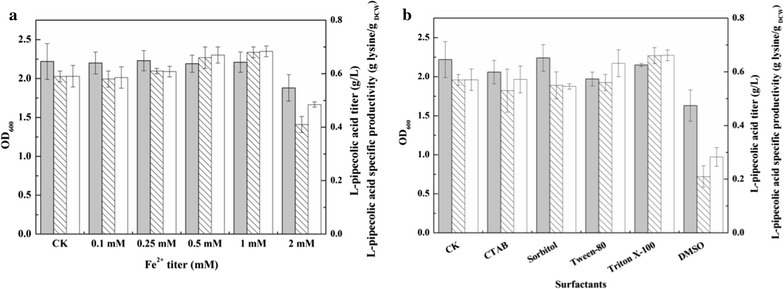
Effect of Fe^2+^ and surfactant on l-pipecolic acid fermentation. The *filled bar* represents OD_600_, the *bar with backslash* represents l-pipecolic acid titer (g/L), the *unfilled bar* represents l-pipecolic acid specific productivity (g l-PA/g _DCW_). **a** The effect of Fe^2+^ on l-pipecolic acid fermentation. **b** The effect of surfactant on l-pipecolic acid fermentation

Combining the improved precursor l-lysine flux and rebalanced nicotinamide cofactor content resulted in recombinant strain BL21–*dapA*–*lysC*–*lysA*–*pntAB*–*pipA* that displayed significantly enhanced l-PA production. To further optimize production, fermentation conditions were investigated. According to Tsotsou et al., Fe^2+^ can stimulate the LCD enzyme activity by 13-fold [28]. However, excess metal ions might inhibit enzyme activity or cell growth as well [19]. Therefore, in the present study, we investigated the effect of the Fe^2+^ concentration on BL21–*dapA*–*lysC*–*lysA*–*pntAB*–*pipA* fermentation. As shown in Fig. 5a, the l-PA titer increased with the increasing Fe^2+^ concentration between 0.1 and 1 mM, but inhibition was observed at 2 mM, at which the OD_600_ and l-PA titer decreased 16.4 and 29.3% respectively in comparison with CK. Supplementation with 1 mM Fe^2+^ increased the l-PA titer to 0.68 g/L.

In the fermentation process, metabolites are produced intracellularly and excreted from cells via transporters, but no transporter of l-PA has been identified to our knowledge. Nevertheless, through our previous studies, excessive l-PA might inhibit LCD activity [19]. Thus, the transport of l-PA should be improved. In general, the barrier permeability of cells can be altered, and the mobility of intra/extracellular substrates and products can be improved upon the addition of surfactants during fermentation. In the present study, five commonly used surfactants were tested (Fig. 5b). While no obvious change in l-PA titer was observed upon addition of CTAB, sorbitol, or Tween-80, supplementation of 1 mM DMSO decreased the l-PA titer by two-thirds to only 0.20 g/L. However, an l-PA titer of 0.64 g/L was obtained upon addition of 1 mM Triton X-100, which was 12.3% higher than in CK, indicating an improvement in the mass transfer of l-PA through the cell membrane.

### Enhanced l-PA production by fed-batch fermentation

**Fig. 6:**
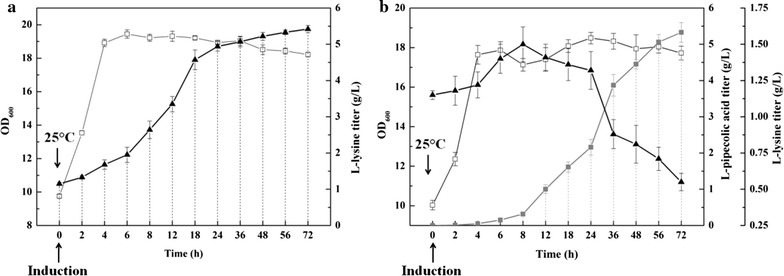
Enhanced l-pipecolic acid production via fed-batch fermentation. The *unfilled square* represents OD_600_, the *filled triangle* represents l-lysine titer (g/L), the *filled square* represents l-pipecolic acid titer (g/L). **a** The l-lysine production using BL21–*dapA*–*lysC*–*lysA*–*pntAB* via fed-batch fermentation. **b** The l-pipecolic acid production using BL21–*dapA*–*lysC*–*lysA*–*pntAB*–*pipA* via fed-batch fermentation

To further characterize the optimized strain and explore fermentation conditions, BL21–*dapA*–*lysC*–*lysA*–*pntAB* and BL21–*dapA*–*lysC*–*lysA*–*pntAB*–*pipA* were cultured in a 7.5 L NBS bioreactor with the addition of 1 mM FeSO_4_ and 1 mM Triton X-100 (Fig. 6a, b). The fermentation process involved a two-phase temperature regime, with the first phase maintained at 37 °C until the OD_600_ reached 8~10 (not shown in the Fig. 6), which usually took 6~8 h with an initial inoculum of 10%. In the second phase, the temperature was adjusted to 25 °C and 0.5 mM IPTG was added to the media to induce over-expression of LCD and lysine metabolism pathway enzymes. The initial glucose concentration of 15 g/L was exhausted shortly after induction, and supplementation of 70% glucose was started straight after to maintain the residual glucose concentration as ~5 g/L.

As shown in Fig. 6a, the OD_600_ of the BL21–*dapA*–*lysC*–*lysA*–*pntAB* increased continuously after the cold induction and finally reached 19.4 with a lysine titer of 5.45 g/L after a 48 h induction and maintained till the end of the fermentation. On the other hand, with an over-expression of LCD in the strain BL21–*dapA*–*lysC*–*lysA*–*pntAB,* the biomass was slightly less (OD_600_ = 18) after a 5 h induction, suggested overexpression of LCD was not overly harmful to cell growth. In this strain, the lysine concentration increased between 0 and 8 h after induction, then decreased as a l-PA accumulated after 8 h. After 36 h, l-PA production slowed slightly, presumably due to lower precursor l-lysine availability, which declined gradually after the exponential phase (Fig. 6b). The highest l-lysine concentration (1.5 g/L) was observed at 8 h, but this remained below 1 g/L during the second half of the fermentation, indicating a relatively high lysine consumption rate and efficiency. With a total supplement of 120 g (40 g/L) glucose, the l-PA titer and yield reached 5.33 g/L and 0.13 g/g glucose, respectively. The sum of l-lysine production and l-PA production was higher than production of l-lysine alone in the fed-batch process, which may be explained by a metabolic pull effect [29].

## Discussion

The production of single enantiomers of chiral compounds or intermediates has become increasingly important in the pharmaceuticals industry [1, 2]. l-PA, an important chiral precursor for the synthesis of optically pure piperidine rings, forms core structure of many naturally occurring alkaloids and drugs in clinical use [9, 18]. l-PA can be biosynthesized using crude enzymes or whole-cell biocatalysts [14, 15, 19], and several of the different catalytic steps involved have been investigated, including some that are unstable and accompanied by high manufacturing costs that limit their applications. However, few researchers have focused on the direct fermentation of l-PA, even though fermentation potentially offers an alternative, efficient, low cost method [22]. In the present study, we expanded the *E. coli* metabolic pathway by introducing a heterologous lysine cyclodeaminase that can biosynthesize l-PA directly from glucose. Furthermore, by improving the precursor l-lysine flux and rebalancing the intracellular cofactor concentration, a competitive l-PA titer was achieved. To our knowledge, this is the first fermentative production of l-PA in a recombinant *E. coli* host.

Wendisch et al., previously attempted to develop the l-PA producers by engineering the industrial lysine producer *C. glutamicum* to fermentatively synthesized l-PA from glucose via the P6C pathway through heterologous expression of lysine dehydrogenase (*lysDH*) and pyrroline 5-carboxylate reductase (*proC*) [22]. Although they fully optimized the glucose uptake system and achieved an acceptable level of by-product elimination, the l-PA titer, yield and productivity were low (below 1.9 g/L or 15 mM, 0.1 g/g and 0.05 g/L h, respectively). The major factor may be the complicated metabolic process involved in the conversion of l-lysine to l-PA, which employs two enzymes and an spontaneous reaction. To further complicate matters, the two enzymes require both NAD^+^ and NADPH co-factors. In addition to the P6C route, however, lysine cyclodeaminase offers an alternative method for fermentative l-PA production. Advantages include fewer enzymatic steps and thus the potential for faster growth of the recombinant strain and more efficient protein production. Additionally, LCD might be more efficient at converting lysine in vivo and therefore lowering the intracellular concentration to alleviate potential feedback inhibition.

For the efficient biosynthesis of the l-PA, a high metabolic flux of the direct precursor l-lysine is important. Diverse l-lysine biosynthetic pathways exist among different species [29]. In the bacteria, l-lysine is derived from l-aspartate via the DAP pathway. In different bacterial species, the first steps (*dapA*) and the last step (*lysA*) are shared when l-lysine was synthesize from l-aspartate semialdehyde. There are three pathways associated with these steps: a succinylase pathway involving succinylated intermediates, an acetylase pathway, and a dehydrogenase (*ddh*) pathway. *C. glutamicum* has both the succinylase and dehydrogenase pathways, whereas *E. coli* possesses only the former pathway. In the present study, four genes (*dapA*, *lysA* and *lysC* from *E. coli,* and *ddh* from *C. glutamicum*) were evaluated for their lysine synthesis ability. The combined over-expression of *dapA*, *lysA* and *lysC* provided the highest lysine accumulation. However, integration of the more efficient dehydrogenase pathway into *E. coli* did not increase l-lysine and l-PA production in the engineered strain, similar results were obtained in previous researches reported by Lee et al. and Cremer et al. [29, 30]. They noted that the differences in nitrogen affinity between the two pathways affected the relative flux and suppressed the ddh pathway. We also hypothesis that the less efficiency of the ddh pathway might be due to the limited supply of the upstream precursor oxaloacetate. Further metabolic engineering of central metabolic pathways might solve this problem.

The catalytic efficiency of LCD has been described as poor by various researches [22]. For example, Wendisch et al. noted that in comparison with the P6C pathway, the relatively low turnover number of LCD may limit l-PA biosynthesis. Previous chromatographic analysis of the denatured LCD by Walsh et al. indicated that LCD is initially folded into three forms: apo, NAD^+^-bound, and NADH-bound. However, only 1/6 of the LCD was in the NAD^+^-bound form and possessed fully activity [18]. Since the cofactors are incorporated during the protein folding process (our unpublished work), rebalancing the nicotinamide pool may improve the intracellular NAD^+^ concentration and its incorporation into LCD. The *pntAB* enzyme uses the electrochemical proton gradient as a driving force for the reduction of NADP^+^ to NADPH by oxidation of NADH to NAD^+^, and this is linked to many systems. For example, Bott et al. heterologously expressed the PNTs in the *C. glutamicum,* which enhanced the l-lysine formation [31], and Xu et al. over-expressed the native PNTs in *E. coli* to enhance the in situ regeneration of NAD^+^-NADPH [26]. In the present study, over-expression of PNTs also stimulated the efficient conversion of NADP^+^ and NADH to NAD^+^ and NADPH, which resulted in a 22.7% increase in NAD^+^ concentration and a 35.9% improvement in the l-PA titer. However, a 42.4% decrease in the NADPH concentration was also observed, but even though 4 mol of NADPH are required per mol of lysine in the lysine biosynthesis pathway starting from oxaloacetate, this decrease in the NADPH concentration did not significantly influence the l-PA accumulation in the recombinant *E. coli* strain. This may be due to several reasons: firstly, lysine pathway genes *dapA*, *lysC* and *lysA* are not NADPH dependent, and thus not sensitive to a decrease in NADPH; secondly, since two more supplementary NADPH pathways are present in *E. coli*, the efficient in vivo conversion of l-lysine to l-PA might involve pulling metabolic flux through the l-lysine biosynthesis pathway using NADPH from alternative sources to alleviate the NADPH deficiency caused by PNTs. In particular, expression of PNTs might improve the uptake of carbon sources, which might also benefit final product formation [31]. In addition to increasing the intracellular NAD^+^ concentration, Fe^2+^ is reported to stimulate LCD, and exogenous supplementation was therefore investigated [28]. Metal ions play a crucial role in protein folding and stabilization, as well as the transfer of electrons. Among the many transition metals, only iron and copper are used in electron transferases. Given that the tightly bound NAD^+^ of LCD may be difficult to rotate during the complex reaction process [18], a possible role for Fe^2+^ might be to assist the electron transfer procedure in this protein. However, the detailed mechanism of the role of Fe^2+^ requires further structural and computational evidence. Supplementation of 1 mM Fe^2+^ was selected because excess Fe^2+^ would be harmful to cell growth.

Transport of target products through the membrane can benefit from metabolic engineering to improve export and increase the volumetric productivity. For example, *lysE* is responsible for lysine export, and its over-expression can enhance lysine production in the lysine producing strain [32]. However, the l-PA transport process is still poorly understood. An alternative way to improve product transport was therefore attempted by adding Triton X-100 as a surfactant, and a concentration of 1 mM was optimal and increased the l-PA titer by 17.2%. Fed-batch cultivation of BL21–*dapA*–*lysC*–*lysA*–*pntAB*–*pipA* was performed in a 7.5 L fermentor, resulting in efficient l-PA production with almost all l-lysine converted to l-PA. In contrast, in a previous l-PA fermentation study by Windisch et al., the antagonistic conversion from glucose to l-lysine and l-lysine to l-PA resulted in the incomplete conversion of l-lysine, which remained at nearly 20 mM (2.92 g/L).

## Conclusions

In the present study, we metabolically engineered an *E. coli* strain to efficiently produce l-PA by enhancing l-lysine metabolic pathways, rebalancing the intracellular cofactor concentration, and amplifying the key enzyme lysine cyclodeaminase. Even though the engineered strain excreted 5.33 g/L of l-PA during fed-batch cultivation, further strain improvements may be possible to make this biotechnological process even more competitive. Removal of other lysine degradation and utilization pathways, improving the lysine biosynthetic pool, and enhancing the tolerance of the strain to product accumulation could all lead to further improvements in l-PA production.

## Additional file

**Additional file 1: Table S1.** The summary of strains and plasmids used in the present study. **Table S2.** The summary of the primers used in the present study.
