# Supplementary material for: Expanding metabolic pathway for de novo biosynthesis of the chiral pharmaceutical intermediate l-pipecolic acid in Escherichia coli
Source: Microb Cell Fact. 2017 Mar 27;16:52. doi: 10.1186/s12934-017-0666-0 (PMC5369227; doi:10.1186/s12934-017-0666-0)
Supplement: Supplementary file 1 — Additional file 1: Table S1. The summary of strains and plasmids used in the present study. Table S2. The summary of the primers used in the present study. [file 12934_2017_666_MOESM1_ESM.docx]

**Expanding metabolic pathway for *de novo* biosynthesis of the chiral pharmaceutical intermediate L-pipecolic acid in *Escherichia coli***

Hanxiao Ying*, Sha Tao*, Jing Wang, Weichao Ma, Kequan Chen^§^, Xin Wang, Pingkai Ouyang

State Key Laboratory of Materials Oriented Chemical Engineering, Nanjing 211816, P.R. China

College of Biotechnology and Pharmaceutical Engineering, Nanjing Tech University, Nanjing 211816, P.R. China

^§^Corresponding author: Kequan Chen, e-mail: kqchen@njtech.edu.cn

^§^Corresponding author. TEL.: +86-138-141-80652

* These authors contribute equally to this work.

Hanxiao Ying, E-mail: hxying@njtech.edu.cn

Sha tao, E-mail: taosha@njtech.edu.cn

Jing wang, E-mail: wjing@njtech.edu.cn

Weichao Ma, E-mail: will1015@hotmail.com

Kequan Chen, E-mail: kqchen@njtech.edu.cn

Xin Wang, E-mail: 416558725@qq.com

Pingkai Ouyang, E-mail: pkouyang@njtech.edu.cn

**Supplementary material and methods**

***Strains and plasmids***

Strains and plasmids used in this study are listed in Table S1. *E. coli* Trans-T1 was used for cloning purposes. For expression and biotransformation experiments, *E. coli* BL21(DE3) was transformed with different plasmids following standard procedures [1].

Table S1 Strains and plasmids used in this study.

| Strain or Plasmid | Characteristics | Source or Reference |
| --- | --- | --- |
| Strain |  |  |
| *E. coli* BL21(DE3) | F^–^ompT hsdS_B_(r_B_–m_B_–) gal (λ cI857 ind1 sam7 nin5  lacI lacUV5-T7 gene1), dcm(DE3) | Transgen |
| *E. coli* Trans-T1  *Streptomyces pristinaespiralis* | F^–^φ80 (lacZ) ∆M15 ∆lacX 74 hsdR(r_k_^–^m_k_^–^) ∆recA 1398endA1 tonA  ATCC 25486 | Transgen  This study |
| Plasmid |  |  |
| pCWJ | RSF *ori*, *lac*I gene, Cm^r^, trc promoter | [2] |
| pET-28a | pMB1 *ori*, *lac*I gene, Kana^r^, T7 promoter | Novagen |
| pCDFduet-1 | CloDF13 ori, Sm^r^, *lac*I gene, T7 promoters | Novagen |
| pCWJ-dapA | pCWJ carrying *dapA* gene | this study |
| pCWJ-lysC | pCWJ carrying *lysC* gene | this study |
| pCWJ-lysA | pCWJ carrying *lysA* gene | this study |
| pCWJ-ddh | pCWJ carrying *ddh* gene | this study |
| pCWJ-dapA-lysC | pCWJ carrying *dapA* gene and *lysC* gene | this study |
| pCWJ-dapA-lysA  pCWJ-dapA-ddh  pCWJ-dapA-lysC-lysA  pCWJ-dapA-lysC-lysA-ddh  pET-28a-pipA  pCDFduet-1-pntA-pntB | pCWJ carrying *dapA* gene and *lysA* gene  pCWJ carrying *dapA* gene and *ddh* gene  pCWJ carrying *dapA* gene, *lysC* gene and *lysA* gene  pCWJ carrying *dapA* gene, *lysC* gene, *lysA* gene and *ddh* gene  pET-28a carrying *pipA* gene  pCDFduet-1 carrying *pntA* and *pntB* gene | this study  this study  this study  this study  this study  this study |

The pipA gene was amlified by PCR using the *Streptomyces pristinaespiralis* ATCC25486 chromosomal DNA as the template, the ampliﬁed PCR product was digested with NcoI and HindIII and ligated into similarly digested pET-28a to generate the recombinant plasmid pET-28a-*pipA*. The *dapA* gene, *lysC* gene, *lysA* and *pntAB* gene were amplified by PCR using *E.coli* BL21(DE3) chromosomal DNA as the template. The *ddh* gene (GenBank: BAA13523.1) from *C. glutamicum* was synthesis by Genewiz after an optimization of codons. The linear pCWJ plasmid and linear pCDFduet-1 were amlified by PCR using the circular pCWJ plasmid and PCDFduet-1 plasmid. Next, the amilified genes and linear plasmids were ligated using ClonExpress methods to generated the final plasmids containing desired genes (ClonExpress® II one step Cloning kit from Vazyme). All the primers used in the gene manipulations were listed in the table S2.

Standard techniques were used for restriction analysis, cloning, and agarose gel electrophoresis [1]. Phusion High-Fidelity DNA Polymerase obtained from Takara (Shiga, Japan) was used for all PCR reactions. Restriction endonucleases were purchased from Takara. All primers were purchased from Genewiz (Suzhou, China). Successful cloning was confirmed by sequencing by Genewiz.

Table. S2 Primers used in this study

| Oligonucleotides | Sequences, 5'-3' |
| --- | --- |
| B34-dapA-F  dapA-B34-R  B34-lysC-F  lysC-B34-R  B34-lysA-F  lysA-B34-R  B34-ddh-F  ddh-B34-R  pipA-F  pipA-R  B34-pntAB-F  pntAB-B34-R | tttagagaaagaggagaaatactagatgttcacgggaagtattgtcgc  ctagtatttctcctctttctctaaattattacagcaaaccggcatgcttaagc  tttagagaaagaggagaaatactagatgtctgaaattgttgtctccaaatttggc  gcatgcctgcaggtcgactctagttactcaaacaaattactatgcagtttttgcacc  tttagagaaagaggagaaatactagatgccacattcactgttcagcaccg  gcatgcctgcaggtcgactctagttaaagcaattccagcgccagtaattct  cacacaggaaacagaccatgaccaacatccgtgttgctatcg  gcatgcctgcaggtcgactctagttaaacgtcacgagcgatcaggt  catgccatggagacctgggtcctgg  cccaagctttcagtgggcgggggc  tttagagaaagaggagaaatactagatgcgaattggcataccaagagaac  ttagtggtggtggtggtggtgcagagctttcaggattgcatccac |
| Fuse-pCDF-F  Fuse-pCDF-R  Fuse-pCWJ-F  Fuse-pCWJ-R | ccgctgagcaataactagcataacc  agggagagcgtcgagatcc  tttagagaaagaggagaaatactaggaattcccactagagtcgacctgcaggcatgc  ctagtatttctcctctttctctaaattgttatccgctcacaattccacac |

**Supplementary references**

[1] Sambrook J, Russell DW. Molecular cloning - A laboratory manual. Cold Spring Harbor (NY): Cold Spring harbor Laboratory Press. 2001.

[2] Ma WC, Cao WJ, Zhang BW, et al. Engineering a pyridoxal 5 '-phosphate supply for cadaverine production by using *Escherichia coli* whole-cell biocatalysis. SciRep-UK. 2015;5:15630.
